# From Misdiagnosis to Management: A Case Report of Ocular Inflammatory Disease

**DOI:** 10.7759/cureus.65491

**Published:** 2024-07-27

**Authors:** Catarina M Francisco, Miguel Santos, Sofia Mano, Inês Leal, Joana Coelho

**Affiliations:** 1 Pediatrics, Hospital Sousa Martins, Guarda, PRT; 2 Ophthalmology, Hospital de Santa Maria, Unidade Local de Saúde (ULS) Santa Maria, Lisbon, PRT; 3 Pediatric Neurology, Hospital de Santa Maria, Unidade Local de Saúde (ULS) Santa Maria, Lisbon, PRT

**Keywords:** ocular toxoplamosis, optic coherence tomography, eye infections, pediatric case, pediatrics ophthalmology

## Abstract

Ocular inflammatory diseases encompass a spectrum of conditions characterized by inflammation within the eye, presenting diagnostic challenges and necessitating tailored management. Ocular toxoplasmosis (OT) poses a challenge in diagnosis and management due to its diverse clinical presentations. We present a case report of a 17-year-old female adolescent who presented with blurred vision and ocular pain, initially misdiagnosed as optic neuritis. Despite receiving methylprednisolone, her symptoms persisted, prompting further evaluation. Ophthalmoscopic examination revealed a whitish focus of chorioretinitis adjacent to an old scar, indicative of OT. Optical coherence tomography confirmed retinochoroiditis, and sulfamethoxazole-trimethoprim was added, resulting in improvement.

This case underscores the importance of considering OT in the differential diagnosis of ocular manifestations, especially in individuals with relevant family history, despite atypical presentations. Timely recognition, accurate diagnosis, and prompt initiation of appropriate therapy are crucial to preserve patients’ visual function.

## Introduction

Ocular toxoplasmosis (OT) is caused by the obligate intracellular protozoan parasite *Toxoplasma gondii* (*T. gondii*) and is one of the leading causes of infectious uveitis worldwide [1]. Transmission of *T. gondii* is typically foodborne but can occur from animal to human via oocyst ingestion, from mother to child during gestation, or rarely because of contaminated organ transplantation or blood transfusion [2]. Although the seroprevalence of toxoplasmosis has undergone a progressive 50% decline during the past 20 years, the disease remains a health hazard with social and economic impact in many countries, especially in South America [3].

OT typically presents with uveitis, of which retinochoroiditis is the most common [1]. Toxoplasma retinochoroiditis (TRC) has been estimated to occur in approximately 2% of those infected and is associated with a lifelong risk of recurrence and progressive vision loss [2]. It is less common in childhood than in adults [4]. The diagnosis of TRC is usually made clinically with the identification of moderate to severe vitritis, associated with focal retinochoroiditis, often with an adjacent or nearby retinochoroidal scar [2].

Therapy with sulfadiazine/pyrimethamine and corticosteroids was classically considered standard for TRC [5], though in recent years other antimicrobials such as oral trimethoprim/sulphamethoxazole have been more widely used. For patients who do not tolerate or have contraindications to systemic treatment, an alternative is the intravitreal injection of clindamycin, along with dexamethasone [6], to control inflammation and prevent recurrence. Adequate prophylaxis of recurrence with trimethoprim/sulphamethoxazole [7] is expected to have an effect on the ocular burden of toxoplasmosis [5].

## Case presentation

We present the case of a 17-year-old female adolescent, with no previous medical or ocular history, and a family history of OT (mother). She was transferred from a district hospital with complaints of four days of right eye (RE) blurred vision, ocular pain which was worse with ocular movements, and headache. The best corrected visual acuity (BCVA) was 20/20 for the RE and 20/20 for the left eye (LE). The anterior segment was unremarkable and an apparent marked edema of the nasal portion of the RE optic disc was noted, without hemorrhages or exudates. The neurological exam was normal apart from a dubious RE relative afferent pupillary defect. A diagnosis of optic neuritis was presumed, and the patient was admitted and given intravenous (IV) methylprednisolone (MPDN) for three days. On day 2 of treatment, she underwent cranial and orbital magnetic resonance imaging (MRI), which did not show any relevant changes, specifically no signs of optic neuritis.

**Figure 1:**
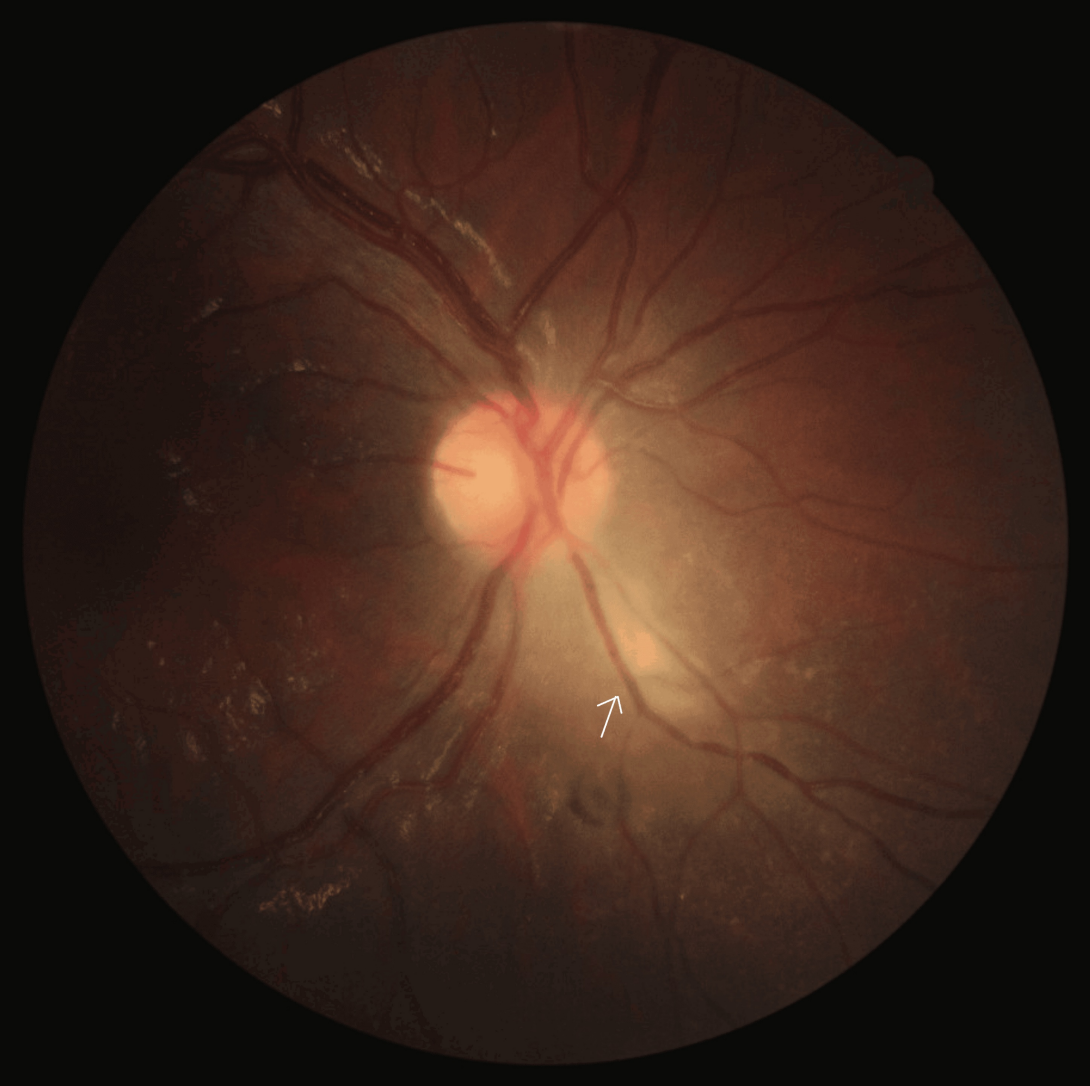
Color fundus photograph of the RE centered on the optic disc taken on day 3 of treatment with IV MPDN. A focal whitish area of chorioretinitis can be seen on the inferior nasal aspect of the optic disc (arrow). A round hyperpigmented scar can be seen adjacent to the lesion. RE: right eye; MPDN: methylprednisolone

Upon re-examination on day 3 of treatment, the ocular pain and headache improved but the remaining symptoms persisted. In the RE anterior chamber, 0.5+ cells were noted with no flare. On fundoscopy (Figure 1), there was only mild effacement of the inferior nasal optic disc border and no clinically evident vitritis but a diffuse whitish area suggesting chorioretinitis was observed immediately adjacent to the inferior nasal aspect of the optic disc, with an adjacent small round hyperpigmented retinal scar which had not been previously detected.

**Figure 2:**
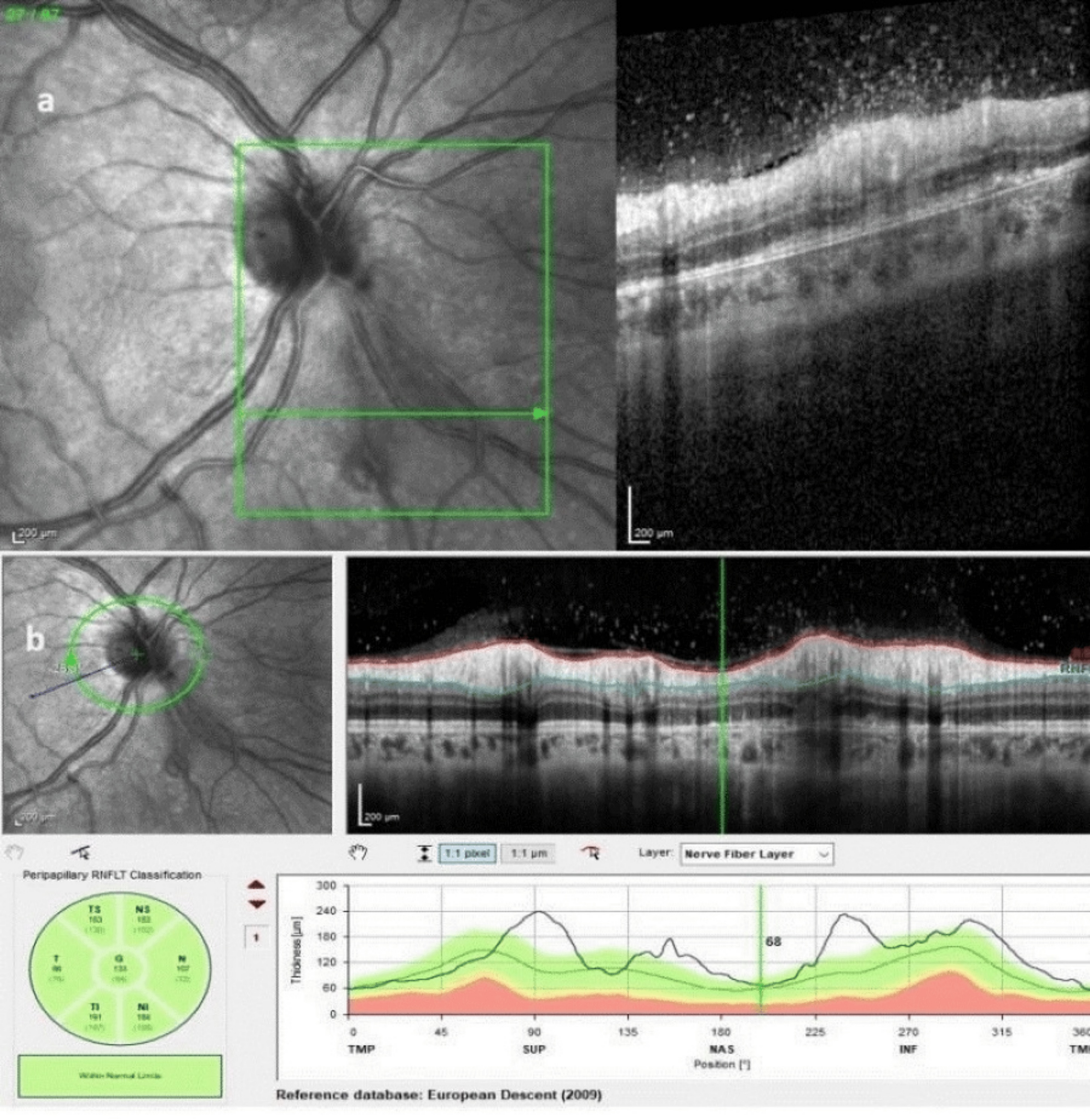
Spectral-domain OCT showing vitreous hyperreflective foci and chorioretinal hyperreflectivity and thickening in the area of the lesion (a) and a slightly increased retinal nerve fiber layer (RNFL) thickness in the nasal inferior aspect of the optic disc (b). OCT: optical coherence tomography

Optical coherence tomography (OCT) demonstrated hyperreflective foci in the vitreous, suggesting vitritis, and increased thickness and hyperreflectivity of the retina and choroid between the optic disc and the scar (Figure 2).

**Figure 3:**
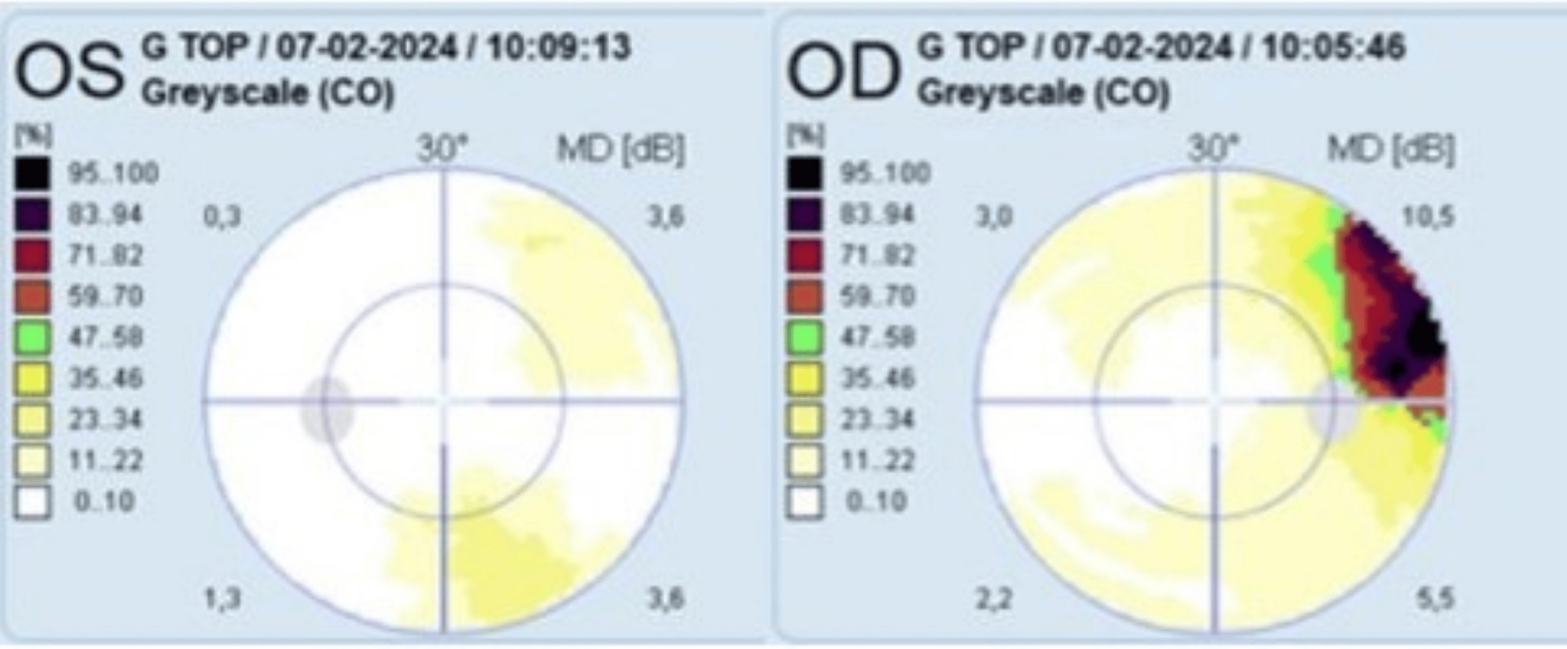
The 24-2 perimetry testing showing a superior temporal visual field defect in the right eye.

The 24-2 visual field test showed a peripheral superior temporal scotoma congruent with the lesion’s location (Figure 3). Serologic testing revealed negative anti-toxoplasma IgM but positive IgG with strong avidity. The remaining bloodwork and serologic testing were unremarkable.

**Figure 4:**
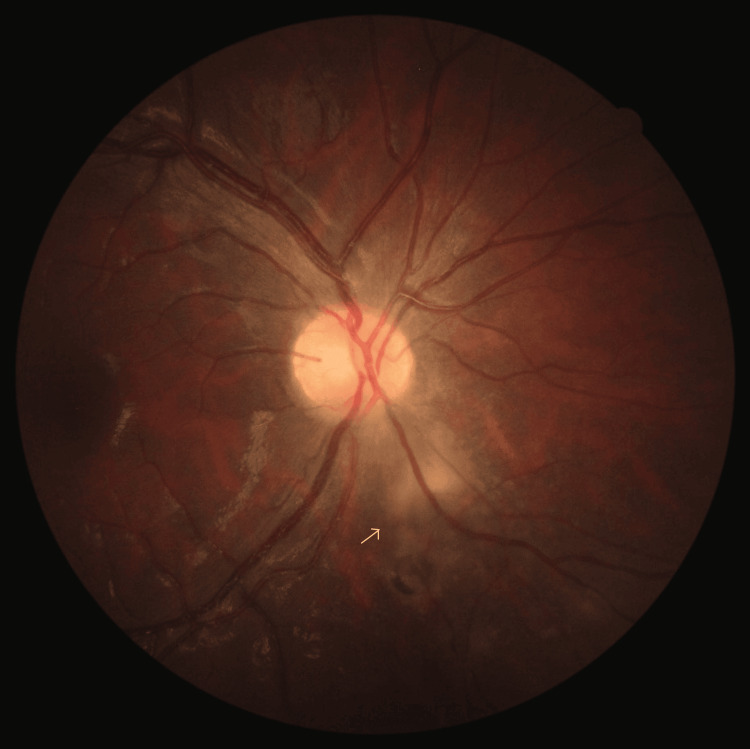
Color fundus photograph of the right eye taken two weeks after the initiation of adequate antimicrobial therapy. An improvement in the activity of the chorioretinal lesion can be seen (arrow).

A diagnosis of OT was then presumed, and the patient was treated with oral sulfamethoxazole/trimethoprim 800/160mg, oral prednisolone at 1mg/kg/day, and topical prednisolone six times per day, with symptom improvement. She was evaluated one week after, still under treatment, with a reduction of lesion size (Figure 4), bilateral 20/20 BCVA, and normal pupillary reflexes.

## Discussion

The presented clinical case shows the importance of the accurate diagnosis of this OT. The erroneous initial diagnosis of optic neuritis due to the location of the inflammatory lesion near the optic nerve led to a delay in the appropriate treatment, as corticosteroids were administered without appropriate antibiotic coverage.

Optic neuritis is an inflammatory optic neuropathy that is commonly indicative of autoimmune neurological disorders [8]. Several cases of toxoplasmic papillitis and juxtapapillary retinochoroiditis have been described in the literature [9,10]. These cases pose a difficult diagnostic challenge with optic neuritis due to often overlapping fundus characteristics and symptoms. A careful history, search for anterior chamber inflammation, and dilated fundoscopy in search of hyperpigmented scars may aid in the differential diagnosis, as was the case in this clinical report. In this case, although there was a suggestive positive family history for OT, as the condition was adequately treated, this hypothesis was excluded. In addition to the evaluation of clinical features, serologic testing may aid the diagnosis of OT as negative IgM and IgG make the diagnosis very unlikely [1].

On the other hand, the presence of high-avidity IgG antibodies against *T. gondii*, even in the absence of IgM, can indicate a reactivation of a latent infection rather than a new infection [11,12]. This serologic profile aligns with the clinical presentation of this patient, who had no recent history of systemic illness but a family history of OT, further supporting the diagnosis.

The serologic tests were performed on this patient only after three days of IV steroids. Although a good clinical outcome was achieved in this case, delayed OT treatment may lead to serious consequences, such as irreversible visual loss [3]. Patients with OT have a lifelong risk of recurrence, which necessitates ongoing monitoring and, in some cases, long-term prophylactic treatment to prevent the reactivation of the disease [13].

In this case, the positive response to treatment and lack of recurrence during the follow-up period are encouraging. However, the patient should be counseled on the potential for recurrence and the importance of regular ophthalmologic evaluations.

## Conclusions

OT is the most frequent cause of infectious posterior uveitis, and the diagnosis depends largely on the recognition of typical clinical findings. Our case underscores the importance of considering OT in the differential diagnosis of ocular manifestations, especially in individuals with relevant family history, despite atypical presentations such as optic neuritis-like symptoms. This case report emphasizes the importance of early recognition, accurate diagnosis, and prompt initiation of appropriate therapy in managing this entity, ultimately aiming to preserve visual function and improve patient outcomes. The visual improvement will depend on the structures involved.
